# Salmagundi

**Published:** 1884-04

**Authors:** 


### SALMAGUNDI.

EDITED BY E. G. BETTY, D.D.S.

Delegates to the American Dental Association will now be appointed by the Executive Committee instead of a special committee as heretofore.

In the discussion on recently introduced remedies, Dr. Howe characterized an antiseptic as a scavenger, and a disinfectant as a policeman. In the heat of the debate we failed to get it in, and it is due the Doctor to insert it here.

When your wife comes into the room and sees you, toothbrush in hand and your mouth bespattered with soapsuds, and lovingly asks: "Scrubbing your teeth, dear?" you take a diabolical delight in answering "No; I'm blackening the stove."

Oxyphosphate of zinc is becoming more popular and entering more largely into general use than anything of a similar nature, plastically speaking, that has ever been introduced, and principally for the reason that it can be used so nearly dry, that pain upon contact is reduced to a minimum.

"I wouldn't be in Egypt," said Mrs. McGill the other day, "for all the wealth of Creosote." Seeing a look of astonishment in the faces of her auditors, she added: "Creosote, you know, was an old Roman god, and everything he touched turned into gold."—*Medical Exchange.*

Dr. Wright referred during the meeting to a short paper read before the Cincinnati Dental Society by Prof. Cassidy, not long ago, and which he was sorry not to have seen published. We asked the Doctor for it at the time, and he promised it to us when he found sufficient leisure to "round it up" for publication.

Dr. Van Antwerp, of Mt Sterling, Ky., of pawpaw fame, was on hand as usual, big, stout, hearty, and jovial as of yore. The combined attractions of the M. V. D. A., Museum, Dog Show, etc., were too tempting to resist. Now, as he was elected President, maybe he will give us a history of the pawpaw digestor by way of an address.

Roasted coffee still holds its own as a disinfectant, and was not mentioned among the list of "remedies introduced during the last three years," because it is older than the most of us, though many don't know how valuable and handy it is. Coffee as prepared for the table is also an excellent antidote for the narcotic effects of tobacco and other noxious *solaneæ;* opium, for instance.

DR. JENNINGS, of Cleveland, was among us again for the first time in several years. He has regained health, strength, and weight, tipping the beam at 189 pounds. He says that he uses a good deal of the Robinson foil, but that it *will* get black when combined with gold and both metals exposed to the fluids of the mouth. When used alone it retains its usual color. This is also our experience with it.

---

DR. BROWN-SEQUARD is opposed to the generally accepted idea that "movements of one-half of the body are innervated from the opposite half of the brain," and says that experiments show "that irritation of one side of the pons varolii or of the medulla, even of the anterior pyramid, causes, eight or nine times out of ten, movements of the limbs on the same side." This may be so; but is it not possible that the irritation, galvanic or mechanical, may have affected only those fibers distributed to that side; or, may not the impulse have reached the periphery by a round-about way?

---

SOME months ago Dr. Watt called attention to the fact that the mainspring of his watch was broken into many pieces, while reposing under his pillow. Maybe the subjoined clipping contains an explanation of the phenomenon:

A scientific jeweler says that fine, sensitive watches are particularly liable to be affected by electrical atmospheric disturbances. During the months of June, July, and August, when these phenomena are most frequent, there are more main-springs broken than during all the remaining months of the year. They break in a variety of ways, sometimes snapping into many pieces. It is also said that since the introduction of the electric light has become so general a large number of watches, some of them very fine ones, have become magnetized. While in this condition they are useless as time keepers.

---

FOWNE says "the vapor of the metal mercury is as transparent and colorless as hydrogen itself," and "is only about seven times heavier than atmospheric air." These facts gleaned from an undisputed authority on chemistry, sustain Prof. Taft in his argument during the recent meeting of the M. V. D. A., that the discoloration of dentine in teeth filled with amalgam is due to the passage of the vapor of mercury into the dentinal tubules and oxydizing there, especially when we remember that for a time the filling hermetically seals the cavity and the temperature raised to 98° or 100° Fahr. in the normal condition, to say nothing of the great increase of heat from beverages daily used; heat, of course, increasing the rapidity of volatilization and the confinement of the filling in the tooth, the power with which the vapor is forced into the dentine.

---

THE following communication to the London *Lancet* of August last seems to indicate that medical practitioners in England are either jealous of the attainments of dentists or afraid that dentistry is encroaching upon foreign ground, and this, too, in the teeth of the recognition by the International Medical Congress, held at London not long ago, of dentistry, by making it a section on the same footing of its other sections and departments:

TO THE EDITOR OF THE LANCET: SIR—I trust you will permit me, through the medium of the *Lancet*, to call attention to a growing evil. It is one of such a nature that if not nipped in the bud it will act disastrously in the future towards the interests of the younger members of the profession. I allude to the practice amongst so-called "surgeon-dentists" of acting in all respects as if they were registered medical practitioners. To quote one instance of many which have come under my notice: A patient of *mine* [italics ours], Miss ———, consulted a dentist for toothache. He informed her that her toothache was attributable to derangement of the liver, and that he would come and see her at her home. Her father objected to this procedure, which, however, did not debar him from sending two bottles of medicine. This is not an isolated case, and I am disposed to think that it is not an idiosyncrasy of London dentists. I am aware that such acts of prescribing are an infringement of the Apothecaries Act, but who is to prosecute? General practitioners cannot unless they desire to expose themselves to obloquy and ridicule. In these days of counter-prescribing chemists, provident dispensaries, grossly abused out-patient departments of hospitals, and an ever-encroaching army of consultants, if to all these we add prescribing dentists, the day is not far distant when the general practitioner will be squeezed out and obliterated.

I am, Sir, yours faithfully,

F. W. East, M. B.

